# Supplementary figures and images for: Cytoplasmic sequestering of a fungal stress-activated MAPK in response to a host plant phenolic acid
Source: PLoS Pathog. 2025 Oct 30;21(10):e1013620. doi: 10.1371/journal.ppat.1013620 (PMC12585099; doi:10.1371/journal.ppat.1013620)

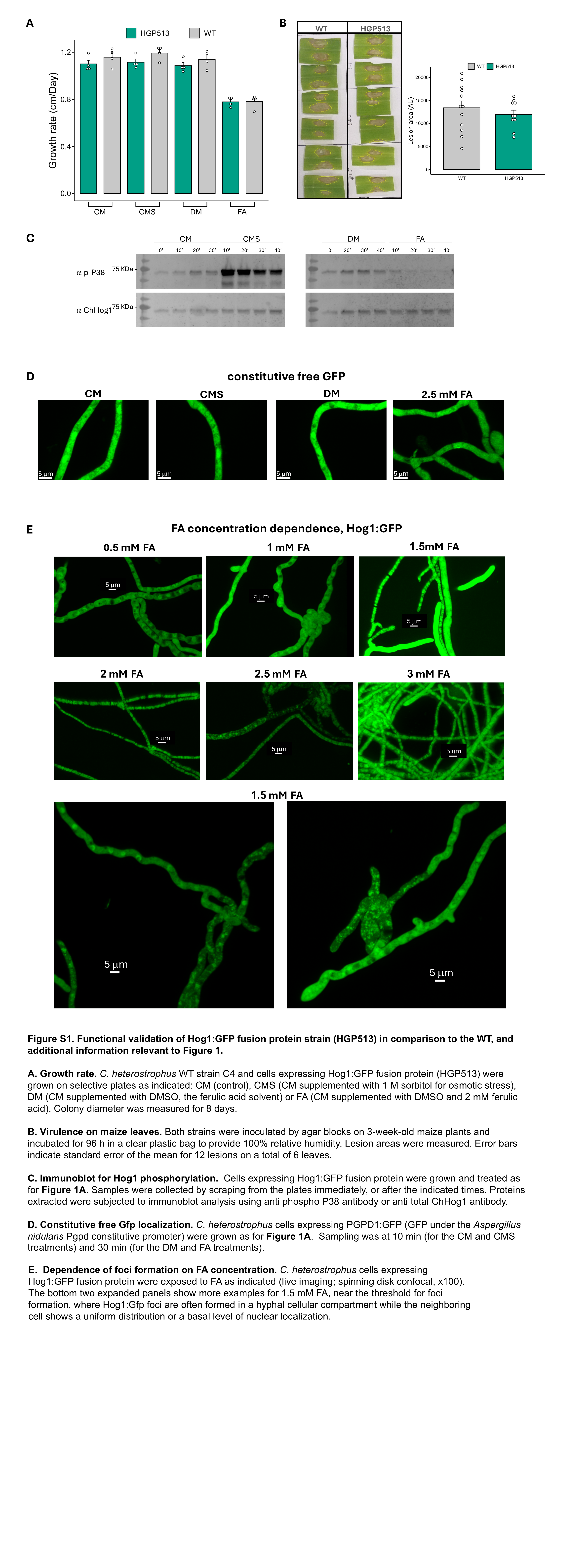

Supplement: S1 Fig — A. Growth rate. C. heterostrophus WT strain C4 and cells expressing Hog1:GFP fusion protein (HGP513) were grown on selective plates as indicated: CM (control), CMS (CM supplemented with 1 M sorbitol for osmotic stress), DM (CM supplemented with DMSO, the ferulic acid solvent) or FA (CM supplemented with DMSO and 2 mM ferulic acid). Colony diameter was measured for 8 days. B. Virulence on maize leaves. Both strains were inoculated by agar blocks on 3-week-old maize plants and incubated for 96 h in a clear plastic bag to provide 100% relative humidity. Lesion areas were measured. Error bars indicate standard error of the mean for 12 lesions on a total of 6 leaves. C. Immunoblot for ChHog1 phosphorylation. Cells expressing Hog1:GFP fusion protein were grown and treated as for Fig 1A. Samples were collected by scraping from the plates immediately, or after the indicated times. Proteins extracted were subjected to immunoblot analysis using anti phospho P38 antibody or anti total ChHog1 antibody. D. Constitutive free Gfp localization. C. heterostrophus cells expressing PGPD1:GFP (GFP under the Aspergillus nidulans Pgpd constitutive promoter) were grown as for Fig 1A. Sampling was at 10 min (for the CM and CMS treatments) and 30 min (for the DM and FA treatments). E. Dependence of foci formation on FA concentration. C. heterostrophus cells expressing Hog1:GFP fusion protein were exposed to FA as indicated (live imaging; spinning disk confocal, x100, scale bars 5 mm). The bottom two expanded panels show more examples for 1.5 mM FA, near the threshold for foci formation, where Hog1:Gfp foci are often formed in a hyphal cellular compartment while the neighboring cell shows a uniform distribution or a basal level of nuclear localization. (TIF) [file ppat.1013620.s001.tif]

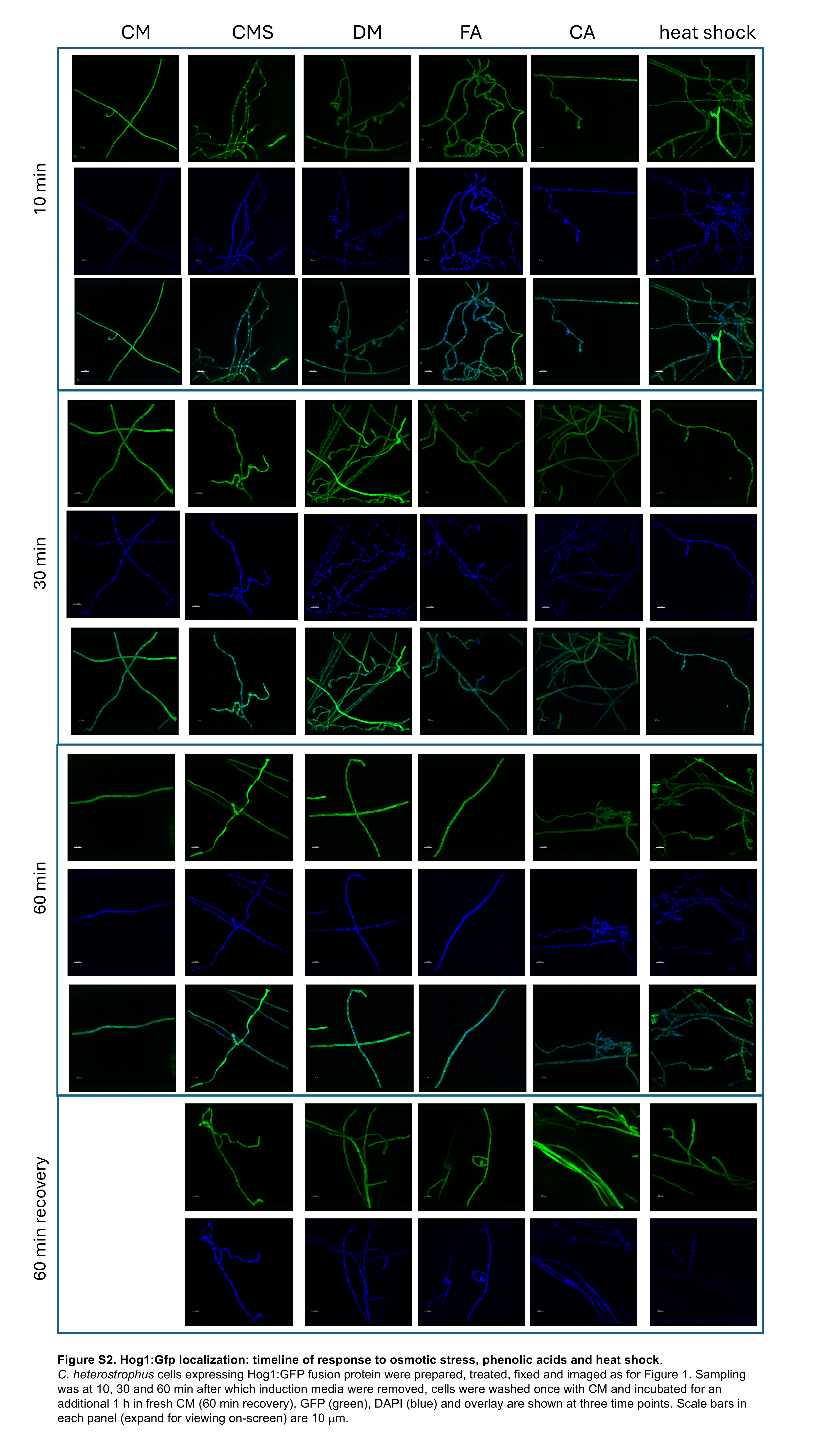

Supplement: S2 Fig — C. heterostrophus cells expressing Hog1:GFP fusion protein were prepared, treated, fixed and imaged as for Fig 1. Sampling was at 10, 30 and 60 min after which induction media were removed, cells were washed once with CM and incubated for an additional 1 h in fresh CM (60 min recovery). GFP (green), DAPI (blue) and overlay are shown at three time points. Scale bars in each panel (expand for viewing on-screen) are 10 mm. At each time point three channels are shown for each treatment: GFP (green), DAPI (blue) and their overlay. (TIF) [file ppat.1013620.s002.tif]

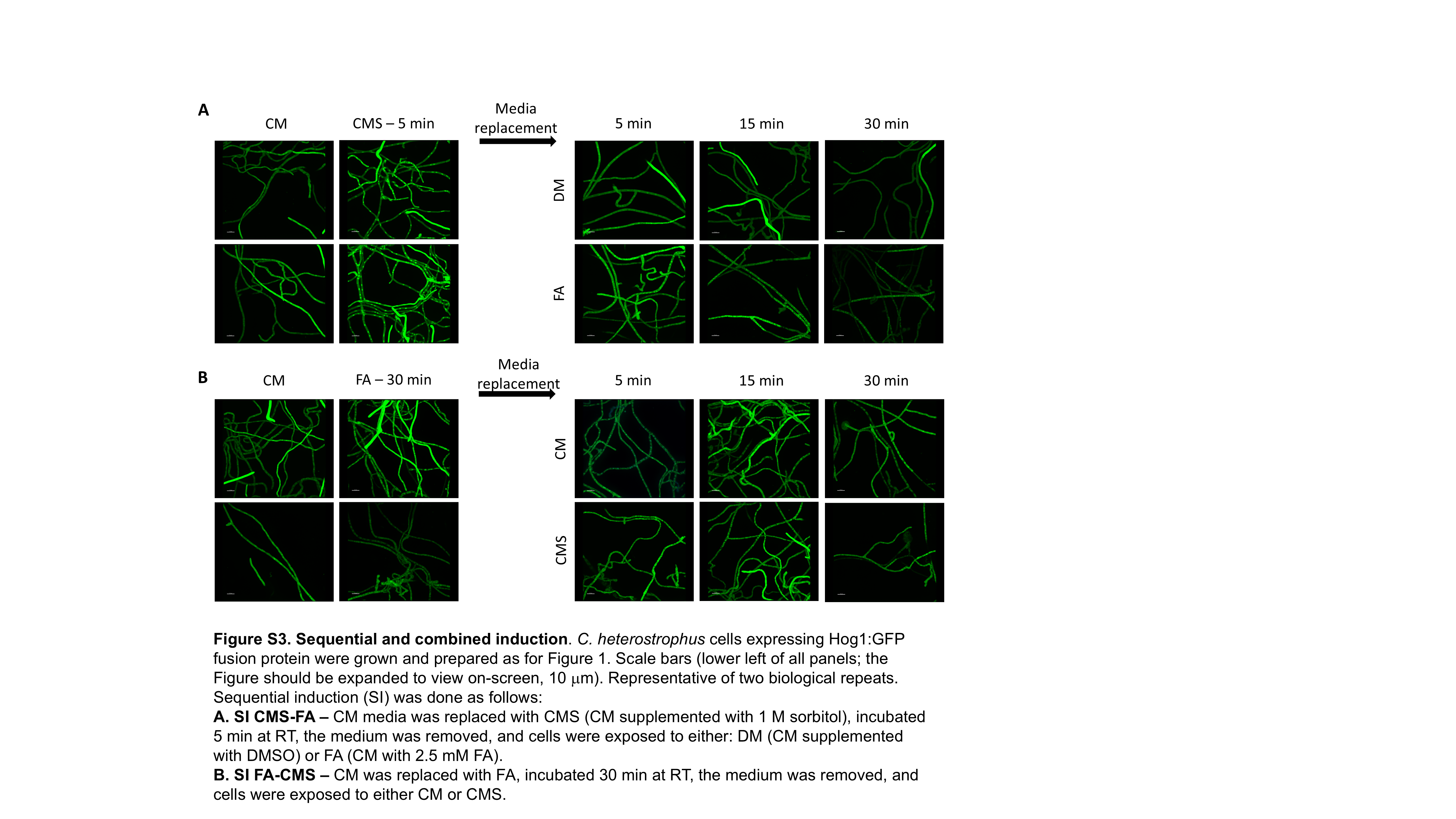

Supplement: S3 Fig — C. heterostrophus cells expressing Hog1:GFP fusion protein were grown and prepared as for Fig 1. Scale bars (lower left of all panels; the Figure should be expanded to view on-screen, 10 mm). Representative of two biological repeats. Sequential induction (SI) was done as follows: A. SI CMS-FA – CM media was replaced with CMS (CM supplemented with 1 M sorbitol), incubated 5 min at RT, the medium was removed, and cells were exposed to either: DM (CM supplemented with DMSO) or FA (CM with 2.5 mM FA). B. SI FA-CMS – CM was replaced with FA, incubated 30 min at RT, the medium was removed, and cells were exposed to either CM or CMS. (TIF) [file ppat.1013620.s003.TIF]

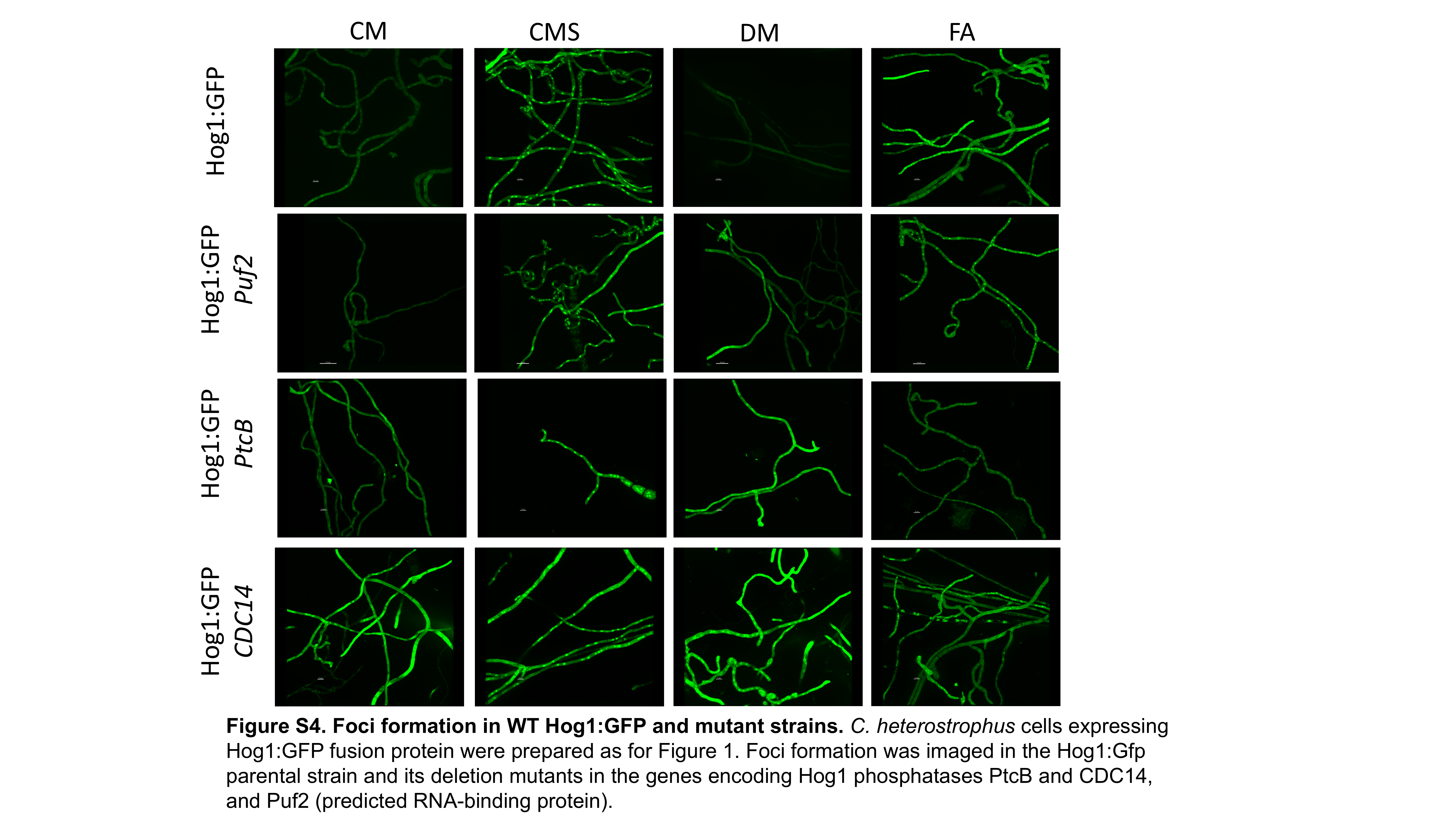

Supplement: S4 Fig — C. heterostrophus cells expressing Hog1:GFP fusion protein were prepared as for Fig 1. Foci formation was imaged in the Hog1:Gfp parental strain and its deletion mutants in the genes encoding ChHog1 phosphatases (PtcB, CDC14) and Puf2 (predicted RNA-binding protein). (TIF) [file ppat.1013620.s004.tif]

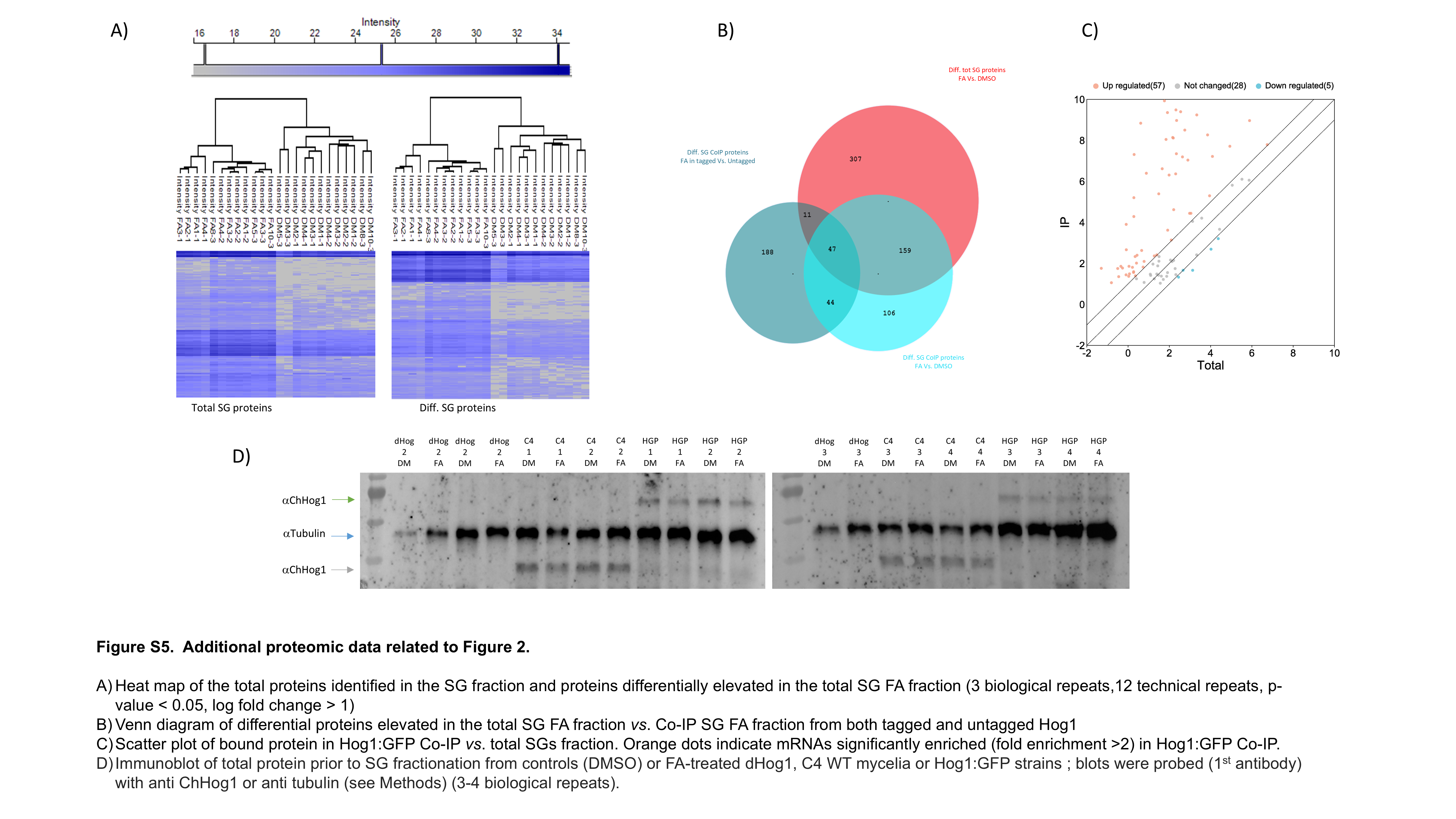

Supplement: S5 Fig — A) Heat map of the total proteins identified in the SG fraction and proteins differentially elevated in the total SG FA fraction (3 biological repeats,12 technical repeats, p-value < 0.05, log fold change > 1). B) Venn diagram of differential proteins elevated in the total SG FA fraction vs. Co-IP SG FA fraction from both tagged and untagged ChHog1. C) Scatter plot of bound protein in Hog1:GFP Co-IP vs. total SGs fraction. Orange dots indicate mRNAs significantly enriched (fold enrichment >2) in Hog1:GFP Co-IP. D) Immunoblot of total protein prior to SG fractionation from controls (DMSO) or FA-treated dHog1, C4 WT mycelia or Hog1:GFP strains; blots were probed (1st antibody) with anti ChHog1 or anti tubulin (see Methods) (3–4 biological repeats). (TIF) [file ppat.1013620.s005.tif]

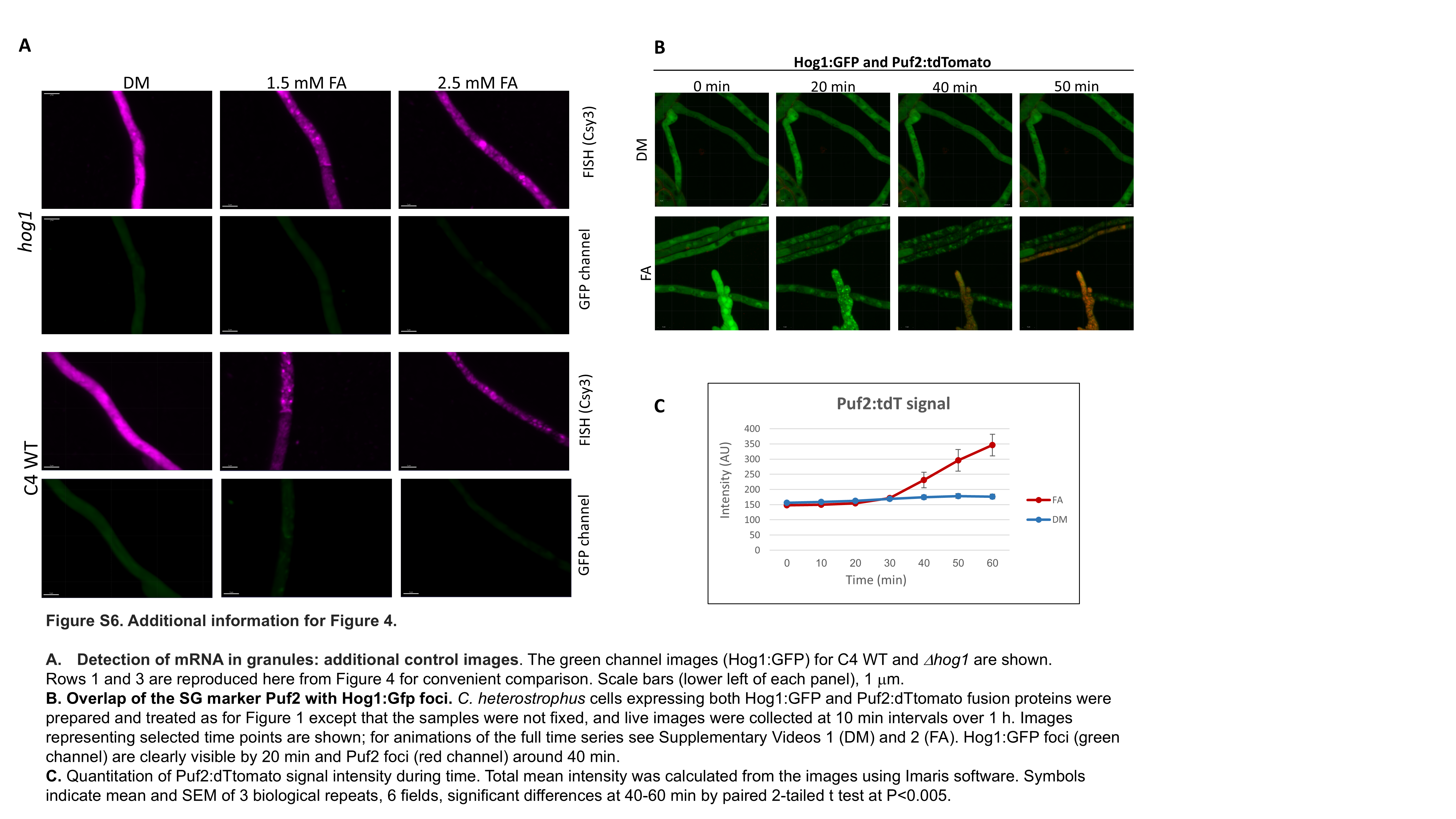

Supplement: S6 Fig — A. Detection of mRNA in granules: additional control images. The green channel images (Hog1:GFP) for C4 WT and Dhog1 are shown. Rows 1 and 3 are reproduced here from Fig 4 for convenient comparison. Scale bars 1 mm. B. Overlap of the SG marker Puf2 with Hog1:Gfp foci. C. heterostrophus cells expressing both Hog1:GFP and Puf2:dTtomato fusion proteins were prepared and treated as for Fig 1 except that the samples were not fixed, and live images were collected at 10 min intervals over 1 h. Images representing selected time points are shown; for animations of the full time series see S1 Video (DM) and 2 (FA). Hog1:GFP foci (green channel) are clearly visible by 20 min and Puf2 foci (red channel) around 40 min. C. Quantitation of Puf2:dTtomato signal intensity during time. Total mean intensity was calculated from the images using Imaris software. Symbols indicate mean and SEM of 3 biological repeats, 6 fields, significant differences at 40–60 min by paired 2-tailed t test at P < 0.005. (TIF) [file ppat.1013620.s006.tif]

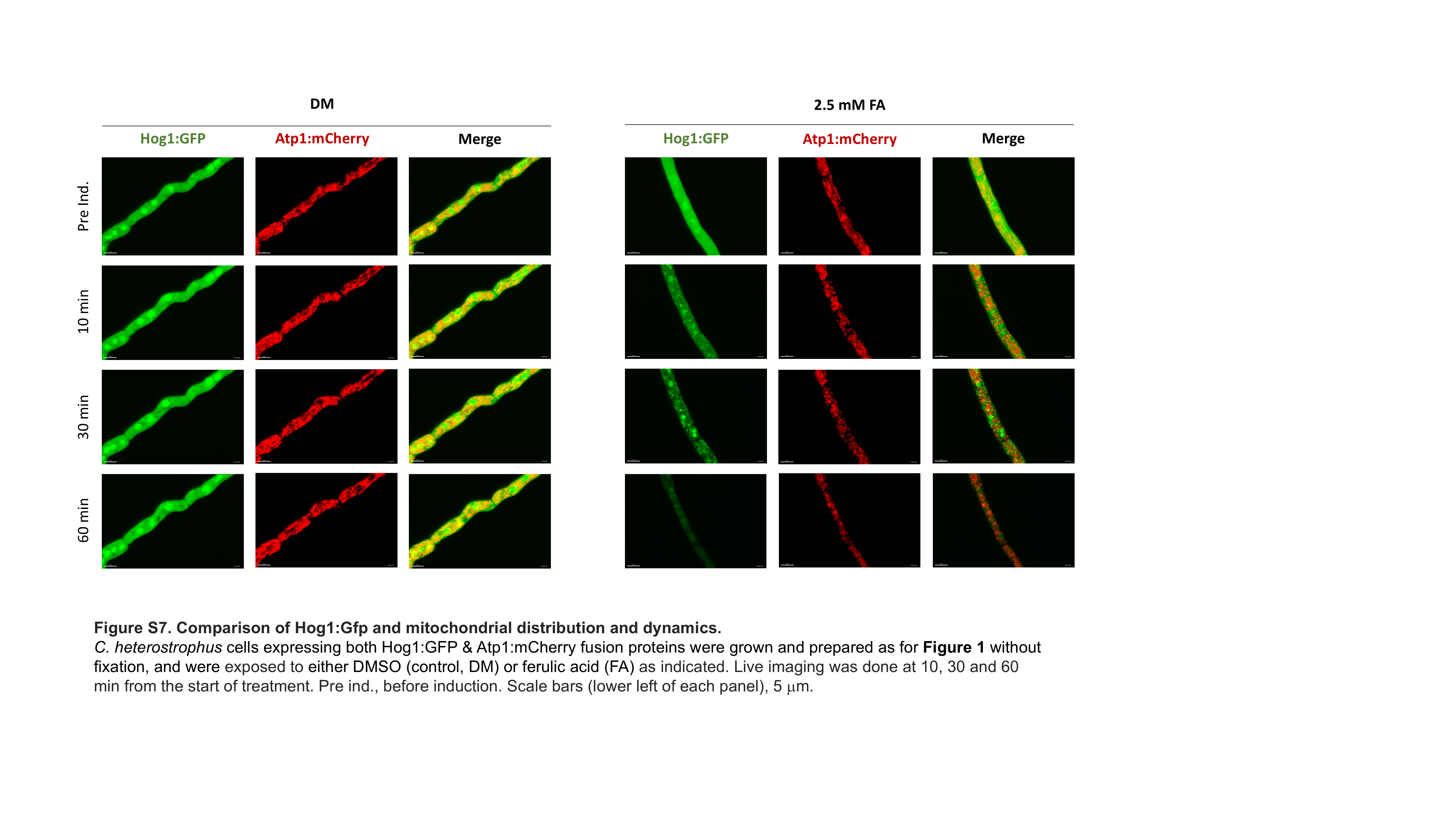

Supplement: S7 Fig — C. heterostrophus cells expressing both Hog1:GFP & Atp1:mCherry fusion proteins, were grown and prepared as for Fig 1 without fixation, and were exposed to either DMSO (control, DM) or ferulic acid (FA) as indicated. Live imaging was done at 10, 30 and 60 min from the start of treatment. (TIF) [file ppat.1013620.s007.TIF]

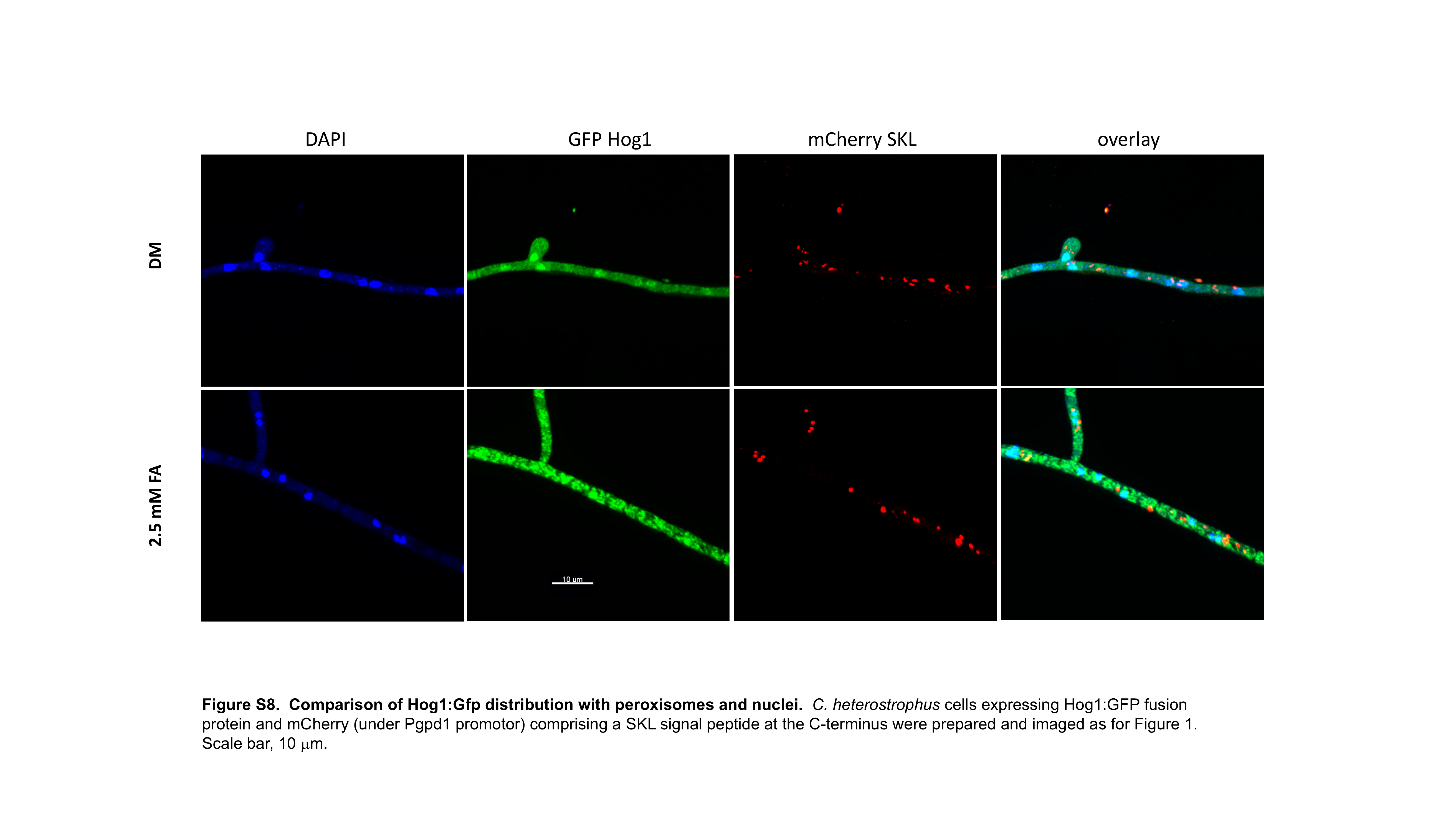

Supplement: S8 Fig — C. heterostrophus cells expressing Hog1:GFP fusion protein and mCherry (under Pgpd1 promotor) comprising a SKL signal peptide at the C-terminus were prepared and imaged as for Fig 1. Scale bar, 10 mm. (TIF) [file ppat.1013620.s008.TIF]

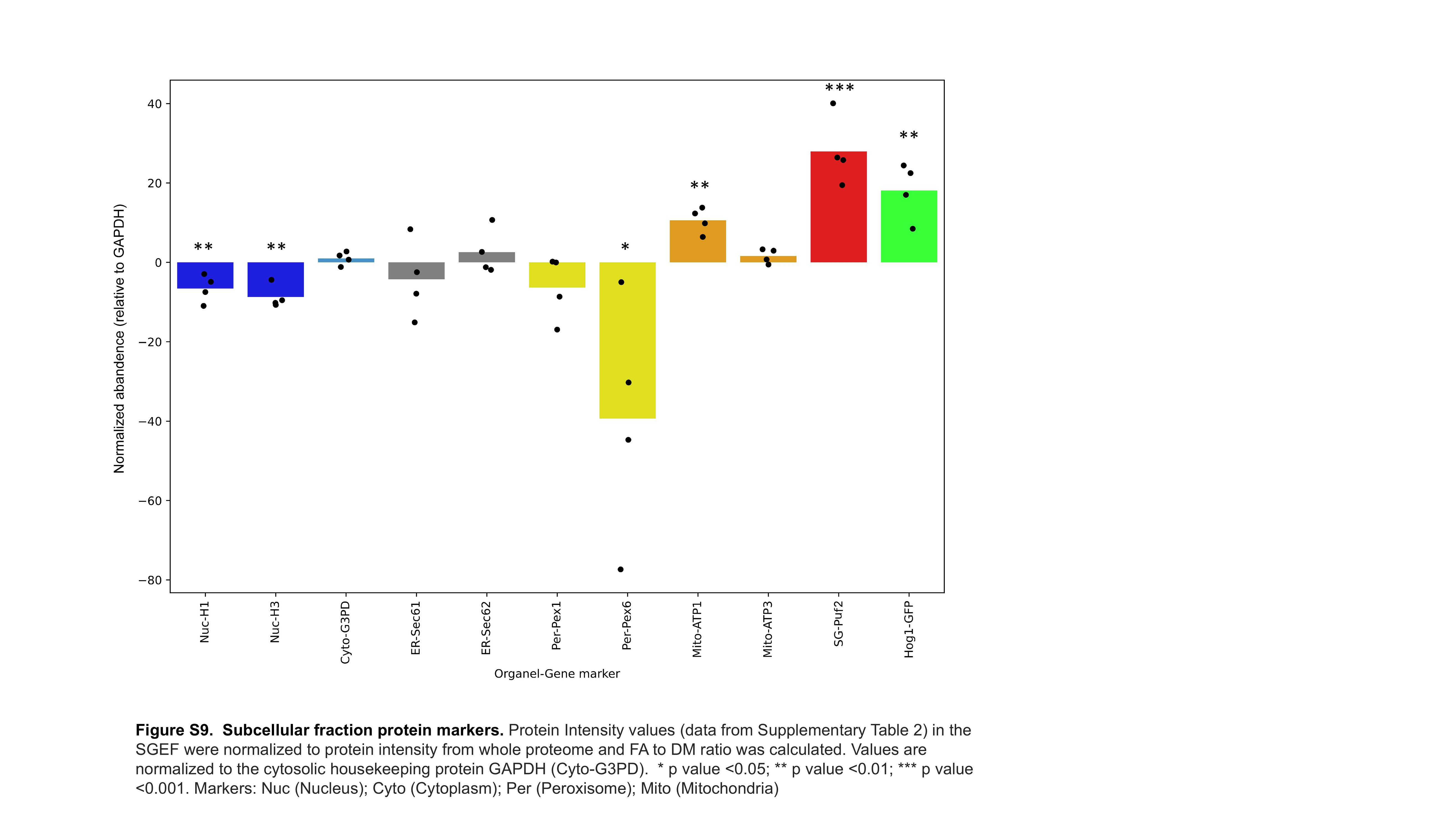

Supplement: S9 Fig — Protein Intensity values in the SGEF were normalized to protein intensity from whole proteome and FA to DM ratio was calculated. Values are normalized to the cytosolic housekeeping protein GAPDH (Cyto-G3PD). * p value <0.05; ** p value <0.01; *** p value <0.001. Nuc (Nucleus); Cyto (Cytoplasm); Per (Peroxisome); Mito (Mitochondria). (TIF) [file ppat.1013620.s009.TIF]

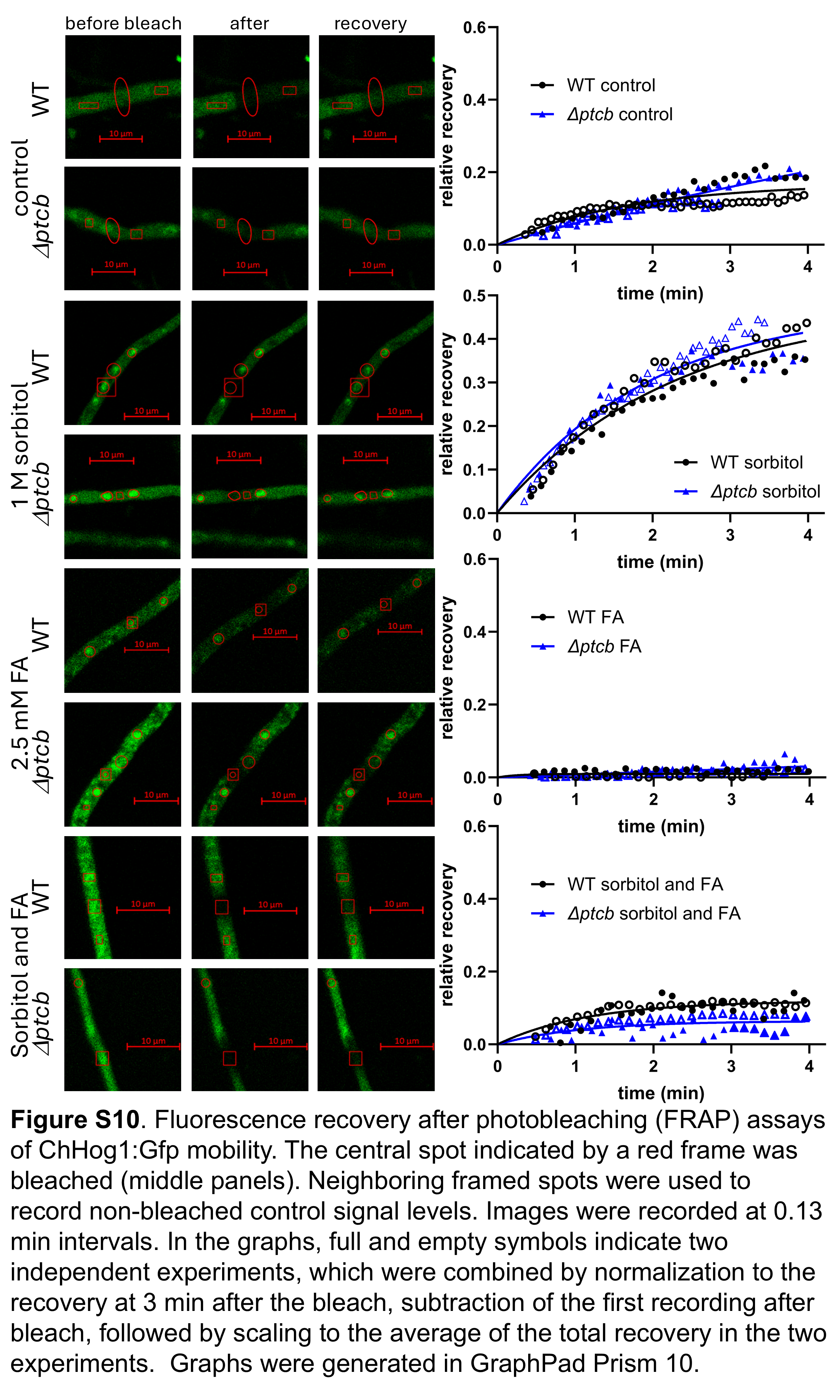

Supplement: S10 Fig — The central spot indicated by a red frame was bleached (middle panels). Neighboring framed spots were used to record non-bleached control signal levels. Images were recorded at 0.13 min intervals. In the graphs, full and empty symbols indicate two independent experiments, which were combined by normalization to the recovery at 3 min after the bleach, subtraction of the first recording after bleach, followed by scaling to the average of the total recovery in the two experiments. Graphs were generated in GraphPad Prism 10. (TIF) [file ppat.1013620.s010.tif]
