# Supplementary material for: Cytoplasmic sequestering of a fungal stress-activated MAPK in response to a host plant phenolic acid
Source: PLoS Pathog. 2025 Oct 30;21(10):e1013620. doi: 10.1371/journal.ppat.1013620 (PMC12585099; doi:10.1371/journal.ppat.1013620)
Supplement: Attachment — Submitted filename: Reviewer Attachments.docx [file ppat.1013620.s015.docx]

The Research comprehensively reports that In maize pathogen Cochliobolus heterostrophus, stress - activated MAPK Hog1 dephosphorylates on exposure to host's ferulic acid (FA). Instead of localizing to the nucleus as in osmotic stress, it forms cytoplasmic foci, colocalizing with mRNA. Mitochondrial fragmentation partially overlaps with Hog1 foci. Using tagged Hog1, an FA - dependent sub - proteome with RNA - binding, translation - related proteins is isolated. Puf2 accumulates and colocalizes with Hog1 foci post - FA induction. Hog1 sequestration to RNA - granules and dephosphorylation may aid fungal survival in the host. Overall, the article is well - organized and presents robust data. Nevertheless, the following areas for improvement need to be addressed:

1. As seen in Figure 1, when only looking at the DAPI staining results, under normal and treatment conditions, the number of nuclei increased significantly after sorbitol treatment. The authors are requested to verify this result. How can this phenomenon be explained? However, after ferulic acid (FA) treatment, there was no significant increase in the number of nuclei. How can this be explained?
2. The authors stated in the article that upon FA treatment, the localization of Hog1 - GFP changes between the nucleus and the cytoplasm. Merely relying on cell localization is difficult to precisely determine this change. It is recommended that in the key results section, a nuclear - cytoplasmic separation experiment be added simultaneously. This would make the results more conclusive.
3. What are the criteria for the authors to select 1 M sorbitol and 2.5 mM ferulic acid (FA)? Does this have an impact on the growth of the pathogen under field concentration? Supplementary controls are necessary to demonstrate that they specifically affect the localization of Hog1 - GFP.
4. Under FA stress, Hog1 foci extensively colocalize with RNA binding protein puf2. Howerver，author further described Hog1 did not colocalize with nuclei.Isn't the RNA-binding protein puf2 located in the cell nucleus? It is reported in the literature that it is reasonable for RNA-binding proteins to be located in the cell nucleus. This conclusion is hard to understand.
5. In the introduction, the background information is comprehensive, but the specific research aim could be more explicitly stated. For example, instead of just listing the known facts about the pathogen's response to ferulic acid (FA), clearly articulate the overall goal of the study, such as "The primary aim of this study is to comprehensively elucidate the molecular mechanisms underlying the dephosphorylation and cytoplasmic sequestration of Hog1 in Cochliobolus heterostrophus in response to FA, and to determine their impact on the pathogen's survival and virulence." This would help readers better understand the focus of the research from the start
6. In the discussion section, more in - depth analysis and comparison of the results with existing literature are needed. When discussing the unique response of Hog1 to FA (dephosphorylation and cytoplasmic sequestration), compare it not only with the response to osmotic stress but also with similar responses in other fungal pathogens under different stress conditions.
7. Lower levels of FA often induced full conversion of Hog1:GFP in a given hyphal compartment to the focal distribution, leaving neighboring compartments unaffected, implying a concerted transition (Figure S1E,F).In this experiment, the author concluded that the aggregation of Hog1-GFP was a peripheral aggregation based on the treatment of low-concentration FA, which cannot be explained by sensation. This is within the pathogen. It is difficult to explain that Hog1-GFP will directly sense the concentration changes in the external environment. The pathogen itself will maintain the dynamic changes within the body.
8. Hog1 is localized to liquid-liquid phase separation (LLPS) condensates upon exposure to FA in vivo.In vitro experiments, will Hog1 undergo protein phase separation? Has the author analyzed whether the Hog1 protein sequence contains disordered sequences?
9. In Figures 1E and F, the protein amounts of Hog1 in different regions were detected to see if there were corresponding internal reference proteins as controls.
10. Co-immunoprecipitation (Co-IP) by Hog1:GFP enriched many proteins including RNA binding protein and mitochondrial proteins,This assays should be called is experiment should be called the Affinity capture mass spectrometry (ACMS). The list of protein data should be listed.
11. The proteins pulled by Hog1 should be further verified. At least, whether puf2 interacts with Hog1's CoIP in vivo should be verified. If it is indeed an interaction, whether FA treatment will increase the interaction between Hog1 and its interacting proteins.
