# Supplementary material for: Cytoplasmic sequestering of a fungal stress-activated MAPK in response to a host plant phenolic acid
Source: PLoS Pathog. 2025 Oct 30;21(10):e1013620. doi: 10.1371/journal.ppat.1013620 (PMC12585099; doi:10.1371/journal.ppat.1013620)
Supplement: Attachment — Submitted filename: point-by-point replies.docx [file ppat.1013620.s017.docx]

**Replies to Reviewers' Comments:**

Reviewer's Responses to Questions

**Part I - Summary**
Please use this section to discuss strengths/weaknesses of study, novelty/significance, general execution and scholarship.

Reviewer #1:  Perhaps a weakness of the study is that the physiological role for this redistribution of Hog1 to cytoplasmic stress granules following FA stress remains unclear.

>The new Figure 5 sheds light on the physiological role of FA in altering the stress response pathway. Specifically, FA suppresses Hog1 hyperphosphorylation, nuclear retention and expression of a downstream gene reporting osmotic stress induciton. Although we had already reported in the first version that FA prevents nuclear accumulation of Hog1:Gfp in response to osmotic stress, the new data in Figure 5 represent a considerable addition to the manuscript.

That said, this is not the same as asking whether the redistribution of Hog1 itself is needed for the action of FA: to answer this question, one would need to block granule formation genetically or with an inhibitor. Despite extensive efforts we have not yet found the means to block granule formation. No gene deletions in the mutants we have studied prevent granule formation, and an inhibitor used in animal cell studies (J. Cell Biol. 2024 doi: [10.1083/jcb.202308083](https://doi.org/10.1083/jcb.202308083)) was not active in our system, perhaps because the sequence of the predicted fungal G3BP ortholog differs in the relevant region.

Reviewer #2: remarks are not classified as major/minor; our replies to all comments follow, in order:

The Research comprehensively reports that In maize pathogen Cochliobolus heterostrophus, stress - activated MAPK Hog1 dephosphorylates on exposure to host's ferulic acid (FA). Instead of localizing to the nucleus as in osmotic stress, it forms cytoplasmic foci, colocalizing with mRNA. Mitochondrial fragmentation partially overlaps with Hog1 foci. Using tagged Hog1, an FA - dependent sub - proteome with RNA - binding, translation - related proteins is isolated. Puf2 accumulates and colocalizes with Hog1 foci post - FA induction. Hog1 sequestration to RNA - granules and dephosphorylation may aid fungal survival in the host. Overall, the article is well - organized and presents robust data. Nevertheless, the following areas for improvement need to be addressed:

1. As seen in Figure 1, when only looking at the DAPI staining results, under normal and treatment conditions, the number of nuclei increased significantly after sorbitol treatment. The authors are requested to verify this result. How can this phenomenon be explained? However, after ferulic acid (FA) treatment, there was no significant increase in the number of nuclei. How can this be explained?

>the number of nuclei is not related to the treatments, rather to the microscope field observed, as the hyphae are multinucleate and nuclei move through the partial septa, as normal in filamentous ascomycetes. In support of this, we have added a note in the legend of Figure 1 to refer the reader also to Figure S2 where there are many examples of DAPI channel images. Although it might be difficult to formally exclude an effect of FA or sorbitol on the cell cycle, one should consider that the treatments are short-term (30 min unless noted otherwise).

2. The authors stated in the article that upon FA treatment, the localization of Hog1 - GFP changes between the nucleus and the cytoplasm. Merely relying on cell localization is difficult to precisely determine this change. It is recommended that in the key results section, a nuclear - cytoplasmic separation experiment be added simultaneously. This would make the results more conclusive.

>upon FA treatment the main change is sequestration within the cytoplasm to subcytoplasmic granules, rather than the nuclear compartment. This is reflected in the comparison of the supernatant *vs* granule fractions, Figure 1 E.

3. What are the criteria for the authors to select 1 M sorbitol and 2.5 mM ferulic acid (FA)? Does this have an impact on the growth of the pathogen under field concentration? Supplementary controls are necessary to demonstrate that they specifically affect the localization of Hog1 - GFP.

>1 M sorbitol is a well-standardized treatment in the literature for osmotic stress. 2.5 mM FA was chosen from concentration series in our previous studies. The local concentration of FA and other phenolics in the maize leaf tissues remains to be determined by microscopic analytical imaging (beyond the scope of this and previous studies); the overall average FA concentration in maize leaves including bound FA is about 0.7 mM. The new Figure 5A includes a concentration series for FA that goes from below to above these values, and shows the impact on growth (albeit in a microtiter plate, not in the field on plants). We recently showed (Levi et al. mBio 2025) that increasing the FA concentration above the endogenous level by spraying on maize leaves decreases virulence of the pathogen, supporting our choice of 2.5 mM as a physiologically relevant concentration.

Under FA stress, Hog1 foci extensively colocalize with RNA binding protein puf2. However author further described Hog1 did not colocalize with nuclei. Isn't the RNA-binding protein puf2 located in the cell nucleus?

It is reported in the literature that it is reasonable for RNA-binding proteins to be located in the cell nucleus. This conclusion is hard to understand.

>RNA binding proteins are not exclusively nuclear-localized, and Puf2 in particular is not nuclear even before addition of FA.

4. In the introduction, the background information is comprehensive, but the specific research aim could be more explicitly stated. For example, instead of just listing the known facts about the pathogen's response to ferulic acid (FA), clearly articulate the overall goal of the study, such as "The primary aim of this study is to comprehensively elucidate the molecular mechanisms underlying the dephosphorylation and cytoplasmic sequestration of Hog1 in Cochliobolus heterostrophus in response to FA, and to determine their impact on the pathogen's survival and virulence." This would help readers better understand the focus of the research from the start.

>the Introduction has been revised and extended, particularly the opening and closing paragraphs.

5. In the discussion section, more in - depth analysis and comparison of the results with existing literature are needed. When discussing the unique response of Hog1 to FA (dephosphorylation and cytoplasmic sequestration), compare it not only with the response to osmotic stress but also with similar responses in other fungal pathogens under different stress conditions.

>the combination of dephosphorylation and sequestration is quite unique so there is no direct precedent in the literature. There is a key paper on Hog1 and (stress) granules in yeasts which was cited and discussed (reference 25, revised manuscript). There is a growing literature from the Neurospora circadian clock on phase separation and signaling and this is also cited. There is a recent paper in the plant literature on phenolic compounds and stress granules that is also relevant (reference 41, end of the discussion).

6. Lower levels of FA often induced full conversion of Hog1:GFP in a given hyphal compartment to the focal distribution, leaving neighboring compartments unaffected, implying a concerted transition (Figure S1E,F).In this experiment, the author concluded that the aggregation of Hog1-GFP was a peripheral aggregation based on the treatment of low-concentration FA, which cannot be explained by sensation. This is within the pathogen. It is difficult to explain that Hog1-GFP will directly sense the concentration changes in the external environment. The pathogen itself will maintain the dynamic changes within the body.

>FA could be the ligand of a fungal receptor, or be taken up and then influence metabolite levels; mM concentrations seem less likely to be detected by a cell surface receptor. The mechanism by which FA is detected will need to be followed in extensive future work beyond the scope of this study.

7. Hog1 is localized to liquid-liquid phase separation (LLPS) condensates upon exposure to FA in vivo. In vitro experiments, will Hog1 undergo protein phase separation? Has the author analyzed whether the Hog1 protein sequence contains disordered sequences?

>There is a small predicted IDR in the C-terminus. *Saccharomyces cerevisiae* Hog1 also has a predicted IDR in the C-terminus, however budding yeast Hog1, in contrast to the fission yeast ortholog, is not recruited to granules upon heat shock (reference 25). In our system, ChHog1 is not required for the formation of mRNA-containing granules (Figure 4) suggesting that Hog1 is recruited to already-formed/forming granules. The reviewer's question is a really interesting one but would need to be addressed with purified recombinant ChHog1 (and compared with other P38 orthologs from yeast, mammalian etc) *in vitro* so we consider this beyond the scope of the current study.

8. In Figures 1E and F, the protein amounts of Hog1 in different regions were detected to see if there were corresponding internal reference proteins as controls.

>the reference protein here is total ChHog1; only the ratios of phosphorylated and total Hog1 signals are compared in Figure 1E (combined from previous 1E and 1F).

9. Co-immunoprecipitation (Co-IP) by Hog1:GFP enriched many proteins including RNA binding protein and mitochondrial proteins,This assays should be called is experiment should be called the Affinity capture mass spectrometry (ACMS). The list of protein data should be listed.

>the complete list of proteins enriched in the Hog1:GFP affinity-captured fraction (ACMS/co-IP) is given in Supplementary Table 2 and deposited in the PRIDE database. ACMS, to our knowledge, doesn't seem to be a routine term in the proteomic literature. We therefore prefer to retain "Co-IP" unless there is a compelling reason to change it.

11. The proteins pulled by Hog1 should be further verified. At least, whether puf2 interacts with Hog1's CoIP in vivo should be verified. If it is indeed an interaction, whether FA treatment will increase the interaction between Hog1 and its interacting proteins.

>we have not claimed to show that Hog1 and Puf2 interact *in vitro* or *in vivo*. There are a large number of proteins in the CoIP/ACMS fraction. FA does increase the enrichment in this fraction (Figure 3) but this does not in itself demonstrate any direct interaction.

Reviewer #3: This study investigates the cytoplasmic sequestration mechanism of the stress-activated MAPK Hog1 in the maize fungal pathogen Cochliobolus heterostrophus under ferulic acid (FA) stress. Through fluorescence labeling, proteomic analysis, and co-localization experiments, the authors demonstrate that FA induces Hog1 to form cytoplasmic foci, which co-localize with RNA-binding proteins such as Puf2 within stress granules (SGs). The study reveals that Hog1 dephosphorylation and sequestration act in coordination, potentially preventing hyperactivation of signaling pathways and thereby enhancing fungal survival in the host environment. The FA-induced formation of Hog1 cytoplasmic foci and their colocalization with RNA-binding proteins, translation initiation factors, and mitochondrial components represents an interesting phenomenon. However, several experimental details require clarification, and mechanistic interpretations of some key conclusions remain incomplete. Additional validation experiments are necessary to enhance the study’s overall credibility.

>point-by-point replies follow

**Part II – Major Issues: Key Experiments Required for Acceptance**
Please use this section to detail the key new experiments or modifications of existing experiments that should be absolutely required to validate study conclusions.

Reviewer #1: Considering the statement in the discussion; FA could be a cue to the pathogen that it needs to contend with host defenses including oxidative or osmotic stresses, both of which raise Hog1 phosphorylation. The observation (Fig S3B) that FA pre-treatment blocks the sorbitol induced nuclear accumulation of Hog1 is intriguing. What would be particularly informative is a western blot looking at Hog1-P over this sequential treatment. Does FA pre-treatment block the sorbitol-induced strong P of Hog1, or does Hog1-P still occur and the FA-induced cytoplasmic sequestering of Hog1 prevents nuclear accumulation.

> the suggested western blot has been added to the new Figure 5

Irrespective of what scenario is correct it would be good to test whether FA pretreatment (and sequestering of Hog1) inhibits or promotes survival following stress treatment (osmotic stress/oxidative stress) that drives the phosphorylation and nuclear accumulation of Hog1.

>done, see the new Figure 5A. As noted above, it is not yet possible to genetically prevent sequestering.

The conclusion that FA dephosphorylation of Hog1 is not a pre-requisite for Hog1 sequestration into cytoplasmic granules is made based upon data presented in Fig. S4B. However, can the authors comment on whether dephosphorylation of Hog1 is important for resistance to FA stress?

>Deletion of phosphatase PtcB prevents dephosphorylation of Hog1 upon exposure to FA. The pleiotropic nature of delta-ptcB makes it difficult to completely separate the different factors, however as previously published (Zuchman et al. 2021 JoF, reference 14/now 15) analysis of growth rates suggests that dephosphorylation is important for resistance to FA stress, as expressed in linear colony growth rate (revised manuscript, end of paragraph 2, Discussion).

Reviewer #2: see replies, above

Reviewer #3: 1. Regarding the abstract statement about Hog1 dephosphorylation being 'surprising,' this observation is in fact consistent with previous findings demonstrating ferulic acid (FA)-induced Hog1 dephosphorylation in C. heterostrophus (Environ Microbiol, 2016, 18:4188-4199). To better understand the functional significance of this response, the authors might consider examining: (1) whether FA triggers Hog1 accumulation in cytoplasmic foci, and (2) whether this dephosphorylation pattern persists during natural host infection. Such experiments could provide valuable insights into the role of this signaling pathway in host-pathogen interactions."

>abstract: corrected as suggested

>(1) FA triggers Hog1 accumulation in cytoplasmic foci – yes, this is one of the main conclusions in the present study

>(2) this would be a very valuable experiment. The antibody we have available is to dual-phosphorylated mammalian P38. It detects the fungal phosphorylated MAPK and likely would detect the plant one(s) as well. We have tried to visualize sequestered ChHog1:GFP *in planta*, and could not obtain sufficient signal intensity and resolution to answer this question. We therefore consider this beyond the scope of the present manuscript, the main aim of which is to report the novel sequestering response, and provide data guiding predictions as to its importance.

2. This manuscript contains multiple significant shortcomings. (1) The introduction section is currently too brief and does not adequately review the current scientific understanding of Hog1 signaling pathways.

>revised, please see the opening and conclusion of the revised Introduction.

(2) Differential interference contrast (DIC) images are missing from all micrographs.

>Added to Figure 1, where the discovery of the sequestering response is first presented. Adding DIC images to all the micrographs would in our opinion burden the already crowded composite figures; the images are available, but we prefer to restrict this addition to Figure 1 as we feel it wouldn't add much relevant information.

(3) Scale bars are absent in Figure 4 and Supplementary Figures S1D-F and others.

>all images are exported with a scale bar, but these are indeed often too small to notice until expanding the figures on the screen, which is often necessary in any case to see the granules or other features. In some cases scale bars were missing from cropped panels, and we have added them, duplicated from the original images.

(4) Figure S2 requires reorganization for clarity, and the DAPI staining is particularly difficult to discern.

>the brightness for the DAPI channel has been uniformly increased over all the DAPI images. The entire composite was reordered so that the three channels of each image appear together, and Figure S2A which summarized conclusions from the images has been deleted and moved to description in the main text.

To directly assess Hog1 mobility between nuclear and cytoplasmic compartments, fluorescence recovery after photobleaching (FRAP) experiments could be performed.

>we have indeed attempted FRAP. The relevant experiment would be to use FRAP to show that the Hog1:Gfp-containing granules are fluid in nature. We could not yet reach the required resolution to do FRAP within the granules, which are rather small; this can be attempted again in the future; at present this is beyond the scope of the manuscript. Mobility between nuclear and cytoplasmic compartments under osmotic stress has been much-studied in the past in other ascomycete models. Our focus here was on granule formation, while data shown for nuclear-cytoplasmic partitioning under osmotic stress serve mainly as a control for the functionality of the Hog1:Gfp fusion protein.

(5) Quantitative analyses (e.g., Fig. 1B and D) lack descriptions of the statistical methods used (e.g., t-test, ANOVA). The overall figure preparation appears rushed.
>descriptions of statistical methods have been added wherever they were missing.

>overall figure preparation: improved; we hope we have not overlooked any major flaws in this revision.

3. The SG-enriched fraction was isolated via differential centrifugation, but the specificity of this fraction is not validated. The authors should perform Western blot analysis to detect known SG markers to confirm successful SG enrichment.

>our SGEF, like its predecessors in the mammalian cell literature, is not specific and includes any subcellular particles pelleted under the same conditions. Hence we refer to "enrichment" rather than "purification" or "isolation." Although antibodies would be available for mammalian cells, we do not have validated SG markers for fungi, though there is support in the literature and from data reported here, for using Puf2 as such a marker. What we did, instead, was to use our proteomic data to confirm successful SG enrichment. These data are provided in Supplementary Figure S9.

4. The co-localization of Hog1 with Puf2 suggests association, but it is unclear whether Hog1 binds RNA directly or is recruited via protein-protein interactions. Additionally, since Hog1 foci still form in the Puf2 deletion mutant, the role of other SG components should be considered and discussed in the context of potential redundancy.

>we have not proposed that Hog1 and Puf2 interact directly. In the proteomic experiments, we followed the approach of the Parker lab (Jain et al. ref. 17) to enrich for the entire granule-associated proteome. The interactions could be tested one by one in future work, and this in our opinion should be initiated following genetic evidence for the functional relevance of a given interaction, for Puf2 and as suggested by the reviewer for other predicted SG components, any of which would require extensive genetic validation and would be a subject for future projects.

**Part III – Minor Issues: Editorial and Data Presentation Modifications**
Please use this section for editorial suggestions as well as relatively minor modifications of existing data that would enhance clarity.

Reviewer #1: It is not clear why so much of the data in the paper is in the Supplementary figures? For example, the immunohistochemistry images shown in Fig S4 beautifully show Hog1 cytoplasmic localisation and dephosphorylated state following FA treatment.

>this was just a question of emphasis and logical flow; following this suggestion we have moved Figure S4 which is now included in the new Figure 5 of the main text. The other supplementary figures in our opinion provide additional validation or support for the main figures. We also note that in the PLoS format the Supplementary figures are very easily accessible to the reader.

For the non-expert, it appears that the localisation of Hog1 demonstrates variation even between different filaments of C. heterostrophus, and in some places the description of the localisation pattern does not seem to match that presented. For example, an aim was to explore if Hog1 could initially be transported to the nucleus upon FA stress. However, in contrast to the conclusion that exposure to FA caused direct Hog1:GFP localization to bright cytoplasmic foci, starting within 10 minutes, in the 10 min FA images there is co-localisation of Hog1-GFP with the DAPI stain (Supp Fig 2)?

>yes, granules and nuclear localization co-exist at the early time points (also for heat shock, Suppl. Fig. S2), we agree with the reviewer and the text has been modified accordingly. We note that there is a variable level of nuclear localization before stress (compare different images from controls not exposed to FA or sorbitol), so one can't conclude (without, perhaps, a rapid-turnover fluorescent construct that was not available in this study) that Hog1 is initially transported to the nucleus, re-exported and then sequestered.

In a further example in Fig S4B, the FA-mediated Hog1 localisation does not look like that in wild-type cells in any of the three mutants tested (Puf2, PtcB, Cdc14). Some comments in the text to support why the authors are confident in their conclusions would be beneficial.

>patterns vary from image to image and our main conclusion here was that none of these deletion mutants lack granule formation. That said, the reviewer's comment has refocused our attention on more subtle phenotypes which would need further work devoted to each specific gene in order to substantiate these phenotypes enough to reach publication stage. Puf2 would be a candidate for such future work. Cdc14 mutants are slow-growing (as published previously). Although some of the *cdc14* hyphal cells shown in Figure S4 look senescent (large vacuoles), nevertheless they formed Hog1:GFP foci upon exposure to FA. To summarize, we agree fully that subtle phenotypes would need more attention, but that the data shown do support our conclusion at the first level, which is that none of these three genes is essential for Hog1:GFP foci formation. The possible interest of the more subtle phenotype of *puf2* is noted in the revised text.

The legends for Supp Fig.1 parts E and F should be aligned to the main legend.
>done

What is the difference between the two FA data points on Supp Fig 1A?
>thanks for catching this typo, the left one is the DM control; corrected.

Figure 1F. The protein samples from the fractionation experiment following control or FA treatment need to be processed on the same blot (currently shown as 2 panels) to allow direct comparison.

>Control and FA samples were indeed processed on the same blot in the one shown in the previous manuscript version, and others used for quantitation. The panels shown had been cropped to show only relevant lanes, and have been replaced by another blot from the series used for quantitation, having adjacent lanes. This being the same set shown in the previous Figure 3E, E and F have now been combined.

Whilst the experiments to explore potential Hog1-GFP co-localisation with mitochondria was supported by Co-IP of Hog1 and mitochondrial proteins, it is not clear why the authors explored whether Hog1-GFP foci colocalised with peroxisomes?
>given that there is no significant colocalization the reviewer's question is certainly a relevant one, but that is in retrospect. We still feel that this was worth testing, using a genetically-encoded peroxisome marker, in view of the hypothesis that ChHog1 is sequestered or docked to RNA-containing granules.

The Discussion is slightly disjointed – and a model figure summarizing the key findings and unanswered questions would be good.

>a model figure seems too premature/speculative at present so we prefer not to add one, but could do so if there is a compelling reason.

Reviewer #2: see replies, above

Reviewer #3: 5. Although Fig. 4A shows Hog1 co-localization with mRNA foci, quantitative analysis is missing. At least three independent biological replicates should be quantified to enhance the reliability of this conclusion.

>quantitative analysis by Imaris software has been added (new Fig. 4B). Though such analysis depends on the cutoff parameters, we agree that this enhances the reliability of the conclusion, while also qualifying it to some extent, as discussed in the revised text.

6. The study does not explore whether Hog1 sequestration affects downstream signaling. e.g., nuclear translocation of Hog1-dependent transcription factors. Expression analyses of Hog1 target genes, particularly oxidative stress-responsive genes, under FA treatment would help clarify functional consequences of Hog1 localization.

> A full study would require RNASeq of WT and mutant strains and this would be for the future; we have done an experiment with a reporter gene for osmotic stress (MST1, see new Figure 5), rather than oxidative stress, as all of the transcripts available to us that could be used to report oxidative stress are ChAP1 downstream targets rather than Hog1 downstream targets. Reviewer 3's suggestion, together with other results now presented in the new Figure 5 led to a new view of the data that puts forward and strengthens the idea that the host defense compound interferes with stress signaling in the pathogen.

7. Fungal strain names should be italicized. Gene and protein nomenclature is inconsistent (e.g., "Hog1" vs. "ChHog1"). The entire manuscript should consistently use "ChHog1" to reflect species-specific gene naming conventions.

>hopefully all instances were addressed in the revision. On the other hand, we feel this is not needed for Hog1:GFP as only as single species/gene/construct is involved, though this could be updated if there is a compelling reason to do so.
