# Supplementary material for: Cytoplasmic sequestering of a fungal stress-activated MAPK in response to a host plant phenolic acid
Source: PLoS Pathog. 2025 Oct 30;21(10):e1013620. doi: 10.1371/journal.ppat.1013620 (PMC12585099; doi:10.1371/journal.ppat.1013620)
Supplement: Attachment — Submitted filename: reviews and replies for R2_oct10.docx [file ppat.1013620.s018.docx]

**Response to Reviewers**

**Reviewers' Comments:**

**Part I - Summary**
Please use this section to discuss strengths/weaknesses of study, novelty/significance, general execution and scholarship.

Reviewer #1: In response to the previously raised concern that the physiological role of FA-induced cytoplasmic foci was lacking, the authors present new data showing that FA treatment prevents activation of the Hog1 SAPK in response to osmotic stress, thus supporting a hypothesis that FA production by the plant may block Hog1 activation during infection and thus could represent a mechanism to inhibit virulence.

Reviewer #2: This study addresses a novel aspect of host-pathogen interactions by uncovering a unique regulatory mechanism of the stress-activated MAPK Hog1 in Cochliobolus heterostrophus (causal agent of maize southern leaf blight) in response to ferulic acid (FA), a key plant phenolic defense compound. Its strengths are notable:

1.Fig. 4B reports “fraction colocalized” but lacks details on statistical methods (e.g., Manders’ overlap coefficient, which is standard for fluorescence colocalization). Adding these values (with SEM and p-values) would quantify the extent of Hog1-mRNA/Puf2 colocalization.

>full details have been added to the Methods. The analysis used spot processing rather than pixel processing, because we are interested in examining the level of interaction between the granules as compartments rather than at the level of individual pixels. In addition, the sub-micrometer dimensions and diffuse borders and overlap of neighboring granules would make the colocalization at the pixel level too noisy. Therefore there is neither a Mander's nor Pearson's coefficient, rather a set of filter thresholds which are now provided in the Methods, so that our analysis can be easily reproduced by others using Imaris software. We have corrected an error in the previous analysis and also added an additional filter. The results of the quantitation have been updated accordingly. This second filter, although named "colocalize spots" in Imaris, is actually an overlap analysis (as was the one in revision 1) and we have renamed the y-axis in line with this. The main text was also updated where we refer to what we see under the microscope, while we continue to mention colocalization when discussing the biological significance of proximity. Furthermore, there is a now new panel in Figure 4B illustrating the spot analysis. The distribution plot in the previous version has been replaced with a bar graph showing the mean and standard error of the fraction of overlapping spots for each direction of the analysis. The total number of overlapping spots no longer depends on the direction of the analysis, however the fraction relative to the number of parent (the channel selected first) spots does, because the number of spots in each parent channel differs between the two directions. The legend to Figure 4B was updated to include all the above changes.

2.Fig. 1E/F conflates “SGEF” and “supernatant” fractions in the legend; clarify fraction names and ensure consistency with the centrifugation scheme (Fig. 1C).

>the relevant text in the legend is now expanded to several sentences, to ensure consistency of fraction names with the scheme in Fig. 1C.

Scale bars in Fig. S1D–F and Fig. 4A are occasionally missing or inconsistent—standardize to 1–5 μm for clarity.

>In Figs. S1 D-F the scale bars generated by the image analysis software were indeed of different sizes in each of the composites (though the same within each composite), so they are now replaced by 5 μm bars.

>In Fig. 4A, all sub-panels contain a 3 μm scale bar. We agree that they are, regretfully, small, and for best viewing require expanding the composite image by zoom-in on the screen. In the previous version of the manuscript additional lettering (3 μm) was already inserted above each bar because the original font was too small. As these scale bars were created directly by the image analysis software, and are the same in all panels so that there is no ambiguity (compare above reply for Figs. S1 D-F), we prefer not to replace them with a different size bar unless really necessary.

3.Experiments to measure Hog1 turnover or retention in SGs (e.g., fluorescence recovery after photobleaching [FRAP] in ptcB mutants vs. wild-type) could test if dephosphorylation affects Hog1’s dynamics in SGs.

>We have done new FRAP experiments to follow Hog1:Gfp dynamics. Under osmotic stress (1 M sorbitol) fluorescence in the nuclei recovers rapidly, consistent with what is known about rapid nuclear-cytoplasmic shuttling of MAPKs. In contrast, when sequestered in granules there is only very slight recovery after photobleaching. Behavior of WT (Hog1:Gfp) and *ptcb* (Hog1:Gfp) strains was similar under both osmotic and FA stresses. These new data are in agreement with our earlier conclusion that once sequestered to granules, Hog1 does not rapidly shuttle between granules and cytoplasm. Furthermore, as FA promotes sequestering in *ptcb* (Hog1:Gfp) (Fig. S4), we had previously inferred that the decreased level of phosphorylation under FA treatment is not critical to sequestering. We've therefore added the FRAP data as a new supplementary figure, S10.

4. Some references (e.g., ref 17: Hsiao et al.) lack complete journal information; ensure adherence to PLOS Pathogens’ reference style (e.g., full journal title, volume, pages).

>ref 17 – the citation is correct as downloaded from the journal website directly into PLoS format; there are page numbers 1-19 in the pdf, however upon opening a few random articles in the same issue, it appears that all pdfs in this epub format are numbered starting at page 1; this is a recurring issue when papers are available as electronic publications and might be acceptable as is?

>following Reviewer 2's comment we have proofread the bibliography, and look forward to working with the editorial office to address any further technical concerns including the one that Reviewer 2 noticed in ref. 17 and elsewhere.

Reviewer #3: The authors have revised and responded well to my concerns. The revised manuscript shows more improved and logical data.

**Part III – Minor Issues: Editorial and Data Presentation Modifications**
Please use this section for editorial suggestions as well as relatively minor modifications of existing data that would enhance clarity.

Reviewer #1: There are still parts of the discussion that are confusing at least to this reviewer.

For example the statement 'FA could be a cue to the pathogen that it needs to contend with host defenses including any stresses that raise ChHog1 phosphorylation' its not clear how FA mediated dephosphorylation of Hog1 and cytoplasmic sequestration alert the pathogen to stresses that require Hog1 activation?

>we reported (ref. 13) that FA induces multiple changes in gene expression, which, in principle, could have signaling roles. That said, this sentence was a remnant of an earlier main hypothesis that dephosphorylation of Hog1 is a direct signaling event, here extend to include sequestering which would also lower the available active Hog1 level. The statement has been rewritten to better align with our current view, and moved to the section discussing the role of Hog1 dephosphorylation and sequestering in the host-pathogen interaction. Please see the tracked copy for additional edits.

Figure S4 is missing details as to which panels represent Wt cells, and those which represent the different mutants.

>our thanks for catching that: when saving the image for the previous manuscript version, the labels were hidden by the microscopy panels; corrected

Reviewer #2: (No Response)

Reviewer #3: (No Response)
